# Key factors leading to reduced recruitment and retention of health professionals in remote areas of Ghana: a qualitative study and proposed policy solutions

**DOI:** 10.1186/1478-4491-9-13

**Published:** 2011-05-21

**Authors:** Rachel C Snow, Kwesi Asabir, Massy Mutumba, Elizabeth Koomson, Kofi Gyan, Mawuli Dzodzomenyo, Margaret Kruk, Janet Kwansah

**Affiliations:** 1 University of Michigan School of Public Health, Department of Health Behavior and Health Education, 1415 Washington Heights, USA; 2 Center for Population Studies, Institute for Social Research; Ann Arbor, Michigan 48109, USA; 3 Ministry of Health, Human Resource for Health Directorate, PO Box M44, Accra, Ghana; 4 University of Michigan School of Social Work, 1080 South University, Ann Arbor, Michigan, 48109, USA; 5 University of Michigan Medical School, Department of Obstetrics and Gynecology, 1500 East Medical Center Drive, Ann Arbor, MI 48109, USA; 6 University of Ghana School of Public Health, PO Box LG 13, University of Ghana, Legon, Ghana; 7 Columbia University Mailman School of Public Health, Department of Health Policy and Management, New York, NY 10032, USA; 8 Ministry of Health, Policy, Planning Monitoring and Evaluation Directorate, PO Box M44, Accra, Ghana

## Abstract

**Background:**

The ability of many countries to achieve national health goals such as the Millennium Development Goals remains hindered by inadequate and poorly distributed health personnel, including doctors. The distribution of doctors in Ghana is highly skewed, with a majority serving in two major metropolitan areas (Accra and Kumasi), and inadequate numbers in remote and rural districts. Recent policies increasing health worker salaries have reduced migration of doctors out of Ghana, but made little difference to distribution within the country. This qualitative study was undertaken to understand how practicing doctors and medical leaders in Ghana describe the key factors reducing recruitment and retention of health professionals into remote areas, and to document their proposed policy solutions.

**Methods:**

In-depth interviews were carried out with 84 doctors and medical leaders, including 17 regional medical directors and deputy directors from across Ghana, and 67 doctors currently practicing in 3 regions (Greater Accra, Brong Ahafo, and Upper West); these 3 regions were chosen to represent progressively more remote distances from the capital of Accra.

**Results and discussion:**

All participants felt that rural postings must have special career or monetary incentives given the loss of locum (i.e. moonlighting income), the higher workload, and professional isolation of remote assignments. Career 'death' and prolonged rural appointments were a common fear, and proposed policy solutions focused considerably on career incentives, such as guaranteed promotion or a study opportunity after some fixed term of service in a remote or hardship area. There was considerable stress placed on the need for rural doctors to have periodic contact with mentors through rural rotation of specialists, or remote learning centers, and reliable terms of appointment with fixed end-points. Also raised, but given less emphasis, were concerns about the adequacy of clinical equipment in remote facilities, and remote accommodations.

**Conclusions:**

In-depth discussions with doctors suggest that while salary is important, it is career development priorities that are keeping doctors in urban centers. Short-term service in rural areas would be more appealing if it were linked to special mentoring and/or training, and led to career advancement.

## Background

The need for human resources in the health sectors of Africa (private or public), has appropriately garnered attention from international policy experts, as well as Ministries of Health throughout the region [1-4]. However, Ministries and donors alike remain uncertain about which, if any, targeted investments have the potential to measurably improve the number, retention and distribution of health personnel [5-8]. Investments have been cautious, and HRH has been described as a potential black hole until interventions have been rigorously evaluated for impact in defined circumstances.

The challenge of developing rigorous HR policy trials in Africa is two-fold: baseline data on unmet needs and priorities of health professionals has not been gathered at significant scale [9,10]; and the existing HR information systems are largely inadequate to measure impact. Data on professional needs and priorities are essential, as professional aspirations change rapidly. While evidence suggests that strategies may require a mix of financial and non-financial incentives, specific reforms and incentive packages require interrogation and evaluation at national level [5].

Ghana's Ministry of Health reports 2442 physicians working in Ghana in 2009 [2]. Sixty-nine percent of doctors practice in hospitals in the Greater Accra region or in the Komfo Anokye teaching hospital in Kumasi, Ghana's second largest city. This distribution is especially disadvantageous for the quality and availability of health care in remote regions of the country. To define feasible policy packages to improve distribution and retention of health workers in rural areas, professional priorities of health personnel in relation to rural service demand investigation [3]. In a recent review of attraction and retention policies, Lehman [7] highlights the need to first analyze local data about health worker decision-making and the challenges of rural service in a given country, in order to inform the selection and packaging of various incentives. The aim of this qualitative study was to gather such data from rural and urban doctors, as well as medical leaders, on both the real and perceived challenges of rural medical service in Ghana. We gathered data from medical leaders across Ghana, and doctors in three diverse regions, to inform the design of pilot interventions that have promise to improve the distribution of doctors in Ghana.

## Methods

This qualitative study is based on 84 in-depth interviews with in-service doctors and medical leaders (Table 1). These interviews were gathered as part of a larger qualitative project involving an additional 114 nurses and nurse leaders in Ghana interviewed at the same facilities (a report on nurses will be reported separately).

**Table 1:** Number of doctors and medical leaders participating in in-depth interviews in Ghana, May-August 2009.

Region	Medical Doctors (no.)	Medical Leaders (regional medical & deputy directors) (no.)	Total
Upper West (UW)	7	2	9
Brong Ahafo (BA)	22	2	24
Greater Accra (GA)	38	2	40
Other 7 Regions of Ghana	Na	11	11

Medical leaders included 17 regional medical directors and regional deputy directors from all ten regions of Ghana. In-service doctors included 67 providers working in three regions: Greater Accra (GA), Brong Ahafo (BA) and Upper West (UW). These three regions were selected to capture the experiences and opinions of doctors working at varying degrees of separation from the urban center of Accra. A complementary discrete choice experiment to assess preferences for rural posting was carried out among senior medical students, and is reported separately [11].

### Selection of doctors

The study undertook a purposeful selection of health facilities, and then requested interviews with available doctors in each facility. Fourteen health facilities were selected in each region (Greater Accra, Brong Ahafo and Upper West), representing all sectors and levels of the health system. The 14 facilities per region included six hospitals of comparable size (approximately 50-bed capacity): two public hospitals, two private for-profit hospitals, and two private not-for-profit hospitals (Mission or Christian Health Association of Ghana [CHAG] hospitals). In addition, in each region we included four mid-sized referral clinics (level b facilities), and four primary health clinics (community-based health planning and services, or CHPS compounds). This sampling plan generated a list of 42 health facilities in total (14 per region), and ensured representation of public and private sector facilities, as well as primary, secondary and tertiary levels of care.

The sampling scheme was executed as planned in Greater Accra and Brong Ahafo, but in Upper West region we learned that there were no private for-profit hospitals. Therefore, in Upper West we included one additional public, and one additional private not-for-profit hospital.

The Ministry of Health sent letters of introduction, clarifying the intent of the study, to each of the three regional medical directors, and to hospital directors. A Ministry of Health representative traveled with the study team at the start of visit to each region, to ensure introductions to the regional directors, who in turn provided introductions to hospital and clinic directors.

All Ghanaian doctors available at a given facility on the visiting day were invited for interview, irrespective of professional rank, as long as they had been in their current post for at least 6 months. A maximum of 6 doctors were interviewed at a given facility but many facilities (especially in remote areas), had fewer doctors in service. In facilities with more than 6 doctors eligible for the study, the first 6 available were interviewed. All interviews were conducted by three team members with experience in qualitative methods. Representatives of the Ministry did not take part in interviews.

### Selection of Leaders

Regional medical directors and deputy directors for the remaining seven regions of Ghana were contacted by telephone; the purpose of the study was described to them, and an interview was requested at a time convenient for them.

Written informed consent was obtained from each participant prior to commencement of each interview. The study was approved by the Ghana Health Service Ethical Review Committee; the KNUST Committee on Human Research, Publications and Ethics; and the University of Michigan Institutional Review Board.

### Interview Guide

A semi-structured interview guide was designed to solicit open-ended discussions on nine themes, identified during successive consultations of the research team and colleagues working in rural Ghana, and review of the literature [2,3,5,8]. These included:

• current conditions of service,

• potential incentives to attract and retain rural clinicians,

• the various understanding and opinions of the current Ministry of Health posting policies, and

• proposed improvements.

Additional questions addressed personal history, motivations, salary, career development, and local amenities. The guide was piloted in Greater Accra and the Northern region; refinements were made prior to commencing the formal study.

### Data

Interviews were carried out over a period of three months starting in May 2009. Interviews typically lasted 30-60 minutes; all were conducted in English, taped, and transcribed verbatim in Ghana. Following an initial read of transcripts, the study team met to discuss both the original and emergent themes. Transcripts were then hand-coded on the agreed dominant themes, and analyzed in duplicate, with each analyst blind to the summary of the other. The team then met to discuss one another's summaries, including other third readers to resolve any differences in emphasis. Overall, analysis and interpretation were characterized by high levels of agreement. Quotes were selected to illustrate majority opinions, unless otherwise noted. Quotes provided in the text are distinguished by italics, and followed by parenthetical notation indicating whether from a medical leader, or if a doctor, by the initials of their region.

## Results

Overall, 67 doctors and 17 medical leaders (total = 84), were interviewed for the current study. Ninety-one percent of doctors, and all leaders who were in the country agreed to an interview (one leader was traveling at the time of the study). The majority of participants were male (87%), ranging in age from 29 to 80 yrs, with a mean age of 36.

Most of the doctors who were currently in rural service in UW or BA were male, and either self-described adventurers, locals from the region who had returned home to serve their communities, or idealists motivated by a mission or ideology. The latter group included Christians who spoke passionately about service for the poor, and socialists who had spent time training in former Soviet countries or Cuba, and who expressed strong commitments to working in the service of health equity and rural development. Whether adventurers, locals or missionaries, most generally described their posting as short-term service to fulfill a personal or nationalistic obligation.

Many doctors in GA had never lived or worked outside of a major metropolitan area like Accra or Kumasi. In fact, while many doctors in GA had been abroad, many (especially the young) had never traveled as far north as Tamale, let alone the upper regions of Ghana.

"I grew up in Accra and lived all of my life in Accra. I was schooled in Accra, from secondary through university. The few times I've travelled have been to the Central Region, and parts of the Volta Region. I don't know anywhere in the Brong Ahafo Region, or the northern part [of Ghana]" (GA).

While some GA doctors expressed interest in serving rural Ghana, this was often mingled with anxiety about the unfamiliarity of rural life, and concern for their career.

### What would it take to make rural service attractive?

All doctors and leaders were asked what the Ministry of Health (MOH) could do, hypothetically, to engage them for three years of service in Tumu, a remote town on the UW border (for those currently posted in UW, the question was how the MOH could make their posting in UW more satisfying). The corresponding responses emphasized three dominant messages (in order of emphasis):

• Provide career development incentives;

• Provide clear terms of appointment, with a reliable endpoint;

• Provide a salary top-up.

Other common responses (but not emphasized by a majority of respondents), included clinical infrastructure (mentioned most often by those currently in GA), especially equipment; ensuring adequate accommodation; and provisions for the schooling of children.

### Career Development

Career development was identified by an overwhelming majority of doctors and leaders as the most critical disincentive for doctors to work in remote or rural postings. An overarching theme from all these interviews was that opportunities for career mobility and further training are currently structured to favor those working in Accra or Kumasi, and to hinder those who work in the periphery. Doctors from all regions describe Accra and Kumasi as the best places to access specialist training, study leave or international opportunities, and the places where one has the best chance to receive mentoring by specialists and senior doctors.

### Lack of Rewards or Recognition

Doctors and leaders alike stated that the Ministry has failed to offer professional or career incentives for remote, or hardship service. Many emphasized the inherent unfairness in the system, whereby no career advantages were offered for remote service, and instead those who serve in rural posts are actually *under-privileged *for career progression, relative to those who stay in urban centers. Most doctors believe that those who have done housemanship in teaching hospitals have greater success rates on specialty entrance exams (i.e. primaries), and they feel that only by staying in the urban center will you be chosen for new professional opportunities.

Many mentioned the failure by MOH to keep track of doctors, and the tendency for rural doctors serving in remote district hospitals to be 'forgotten" or "abandoned"; this motivates young doctors to stay close to the teaching hospitals to gain recognition.

"One of the reasons why some people don't want to come here [rural Ghana] is because when they want to go back, to specialize or improve their skills [up here] nobody sees them, and nobody will remember them. You are in the district hospital, and the only one who might see you once in a while is if you come in to the regional center, but it's not easy to be picked, to benefit from anything. This is one of the incentives that we need to put in place for those working here." *(Leader)*

Medical leaders were particularly explicit in their frustrations over the speed of promotions within the GHS, and how slow these were when compared to promotions within the teaching hospitals. The irony and illogic of such favoritism was underscored, given that doctors serving in remote services are likely to have more practice and responsibility than those in the teaching hospitals, where the abundance of trainees means less hands-on experience. A leader described two recent graduates of the West African College to illustrate his point: the one appointed to a teaching hospital was quickly appointed as Senior Specialist, while the one with GHS has had endless delays in his appointment.

"But the man I'm talking of is the only gynecologist here [in a remote district], and he works virtually 24 hours because the rest are only housemen. Meanwhile his colleague in Accra is in a team of over 20 people! There are systematic defects in the rewards and promotion system that will continue to attract staff to teaching hospitals if we don't take serious decisions." (Leader).

This unfairness was cited as ultimately promoting those with less experience faster than those with more, with potential consequences for quality of care.

### Lack of Mentoring

Doctors in remote posting were very conscious of their disadvantage when it comes to mentoring and moving up the career ladder, and this was a major source of frustration. Even those who had come north with strong missionary or ideological motives felt that they have now been forgotten by the Ministry, and are at risk of falling off the career ladder.

"Only those who are in the cities have access to the scholarships; if you are in the village it becomes difficult, which shouldn't be the case. Rather it [should] be that when you are in the city it should be difficult to get the scholarship, and when you are in the rural area easier, but things are not done that way"... (UW)

Doctors in the north and remote parts of BA emphasized their professional isolation, figuratively and literally, pointing out that they have no colleagues with whom to share rounds and discuss cases, no colleagues who are easily contactable by phone, and they have to manage the most challenging cases without support or supervision.

"We should have the surgical team but I am the only person here; so the team is just a person, when you are doing rounds you don't have any colleague to turn to; basically you are the captain of the boat, and the only sailor as well."(BA)

Even when a case is beyond their ability, contacting a colleague for advice is often not possible, nor is referral. Patients themselves will not accept referral because the referral facility is far, and they lack means for travel.

"In Upper West... the catchment area is so big, and you are the only person able to serve all these people. You have your specialty area, but end up providing services in areas where you are not fully trained." (UW)

Occasionally a young doctor in GA complained about the lack of hands-on practice in the teaching hospitals, but mentoring in GA was largely characterized in terms of someone to help you move up the professional ladder. Young doctors see the city as the one place to stay on the radar screen(s) of senior doctors who have the power to select or promote them for fellowships, study opportunities, or better appointments. Those in remote postings risk being forgotten and passed over for new career opportunities.

In BA, complaints about a lack of mentoring varied considerably between facilities, as some urban health facilities have multiple doctors and specialists on staff, while other facilities in remote BA are as isolated as those in UW.

"When it comes to mentoring in BA, the regional hospital is okay - that's where the specialists are...but when you move elsewhere, especially to other public hospitals, there's no mentoring." (BA)

There was a notable distinction between the mentoring discussions in CHAG and public hospitals, especially in BA. Several CHAG doctors mentioned that once or twice a year they have expatriate specialists coming to work for a month or so, giving them a chance to learn new procedures. Two regional medical directors mentioned that young doctors have asked pointedly to be posted to specific CHAG facilities because specialists are known to visit there, underscoring the message that specialists provide a magnet for other providers.

### Professional Imprisonment

While doctors in all regions emphasized how hard they work, the loads were characteristically different in urban versus rural settings. Compulsory versus voluntary aspects were crucial. In GA, many doctors said they had a high patient load during their fixed hours, and then they progressed to "locum" work after hours to make ends meet. Despite such burdens, almost all doctors in GA acknowledged that doctors working in remote posts have a heavier load.

The workload in UW, as well as more remote parts of BA, was characterized by what doctors described as the "professional imprisonment" of being the only doctor at the post, and they repeatedly linked this to slower career progression because their hard work didn't translate into any recognition from those with influence, and at the same time the sheer volume of work made it nearly impossible for them to travel to meetings, to network, to study, or pursue new opportunities.

"Sometimes when you come here it becomes difficult to progress; you go back to the teaching hospital and all your colleagues are far, far ahead of you. There is a way for the Ministry to come to your aid. Once you accept to [come] here, if you can serve 2 years, [they should] sponsor you for the next 2 years to study or specialize".. (UW)

"There are doctors in the villages [who] want to go to the college, maybe to Korle-Bu to specialize, but they can't because they didn't [yet] pass their exams ...it is not as if they don't think anymore, or can't learn, it's because of the load. Sometimes I'm preparing to do an operation, and you'll find that while they're getting the patient ready, I'm studying." (UW)

"Like I said, the obstacles are that [the doctor] may be there alone, and so leaving to go and do further studies will be a headache for a regional director, because [...] the place is going to be empty. [...] If we lose you we don't know when or how to get somebody else to agree to go there." (Leader)

### No Continuing Education

In UW and BA, many doctors highlighted poor access to the internet, and the absence of library facilities or technical resources.

"Do I even have a learning environment here? I used to have, there used to be the internet at the theatre; sometimes when I close late in the night from 8 to 10, maybe I will sit back and then do a few things, but of late the internet [has been] fluctuating, so learning has been off and on." (UW)

In larger towns of BA and in the capital of UW doctors mentioned that internet was starting to come in, but it was intermittent at best, and not like in Accra or Kumasi.

Leaders emphasized that internet connectivity is progressing quickly, but that doctors and clinical staff are sorely in need of better computer literacy. Several leaders suggested long distance learning as a possible way to meet staff interest in new information and skills.

"I think we should encourage every facility to get an internet connection. And then introduce them to the proper use of the internet. There are a lot of resources but people don't know how to utilize them, and rather use them for unnecessary things." (Leader)

Doctors in UW and BA emphasized that the sheer workload prohibited them from accessing continuing professional development (CPD) credits. When asked about workshops or training seminars, few doctors in UW and BA had been to any in the past 6-12 months, "for the past 7 months, Nil". They emphasized that there are few, if any, CPD credit-earning opportunities in rural areas.

"We're to get 20 credits for re-certification for practice, and that is every year; .... the interesting thing is that ...all the programs are either in Tamale, Sunyani, or Accra; we don't have any centre here for any of our CPD; we always have to travel and we have bad roads; risking our lives we go and get 5 credits." (UW)

"Even the in-service and workshops often happen in the teaching hospitals in Kumasi or Accra, and because of the workload, we can't go, since it's far from here. So we can't take advantage of in-service training." (BA)

For most rural doctors, travel time meant leaving patients without a doctor. They expressed frustration over the implicit advantages to doctors posted at teaching hospitals, who can gain 10 CPD credits on the basis of attending clinical meetings within their own facility.

### Terms of Contract

There is widespread frustration not only about the lack of clear incentives or career development guidelines, but also about ambiguity in contracts. When discussing the basis of postings and terms of appointment, doctors clearly had very uneven information about current policies. Younger doctors were especially uncertain about their terms of contract, or the incentive structures now in use.

"Again, there are no laid down opportunities by the system to say. .'Oh! if you stay here for 4 years, these are the various programs available to you; you are exempted from writing this exam; you can go for a postgraduate program; these things are not clearly defined." (UW)

This creates anxiety and distrust, worsened by widespread concern of being "forgotten" in a rural posting, and concerns that the MOH may not respect the agreed fixed-term contract for service in a remote facility. There were many doctors, in all regions, who feared that once in a remote posting they would have to find their own replacement in order to be transferred.

"There is not enough staff to go around, so if I wanted to leave [the rural post], I would need to find a replacement before moving on, and that is not often easy.." (GA)

"People get to the districts and coming back to the teaching hospitals to do a post-graduate program is very difficult. And again, people are also not very sure; things are not well-defined when it comes to getting to the district and coming back; there may be guidelines but [they are] not known to many people. And even if there are guidelines, they are never implemented because [once] you get to the district, there is no clear cut future; you get to the district and are not sure when is your next move. [..] you have to work your way back, and there's no official system which brings you back to the postgraduate program." (UW)

### Salary

A large majority of doctors and leaders from all regions argued that remote or rural service deserved a higher financial incentive, not only because of the higher workload, but because of the lost opportunity for supplementary income from locum (i.e. moonlighting). Doctors in GA especially emphasized how tough it would be to work without the possibility of locum, and many felt that the salary package simply had to be better to attract doctors into deprived areas, once the loss of locum was factored in. Leaders were confident that salary affected recruitment, but many were not convinced that salary alone would provide adequate pull factors; the dominant opinion was that extra salary should be provided to flatten (i.e. equalize) the playing field, but it was explicit and transparent career advantages that would actually draw people north.

"My colleague at Komfo Anokye gets the same salary as I take here. But they go to work at 8 and then, ... at about 2 they close, so they can go to a private hospital and do locum. But here the whole day you are in the hospital. If they [MOH] also take this into consideration... let me say you make 500 dollars from your locum, [MOH should] give us 700 dollars [in the rural post], as a kind of incentive." (UW)

"Eight doctors were posted here, but none of them came. But there are other people who, if you tell them 'look you go and work around the clock for something, something very substantial', then they will say 'well, let me go and stay for 1 or 2 years of my life'. But [now] I have the same pay as colleagues in Kumasi or Accra; they go to work in the morning and leave at 2, and I work around the clock." (UW)

Another important dimension of salary raised by medical leaders was their frustration that they had so little power to cut-off the salaries of doctors who fail to fulfill their appointments. They complained that the payroll system is unresponsive, and they have limited means to regulate pay for performance. Most insisted that if doctors do not come to their appointed posts they should lose their salary, and if they try to return to Accra and there are no posts available, then they should be forced to opt out of public service.

### Other Incentives

#### Accommodation

Work-based accommodation was a common source of disappointment, arousing complaints among doctors in all three regions. Most doctors had clearly entered the profession with expectations that the MOH, or the private or CHAG health facility, for which they work, would provide or subsidize housing; the reality of inadequate, distant, or nonexistent housing, was widespread. Several doctors in GA mentioned that they (or a friend) had been willing to take a rural post in northern Ghana, but there was no housing available; they would have had to wait months for renovations; these scenarios were offered to explain why they had eventually given up considering a rural post, and settled in Accra. This was affirmed by interviews in UW and BA, where many complaints focused on the absence or inadequacy of units.

#### Hospital Infrastructure

In addition to the above points, doctors stressed the importance of adequate equipment and facilities to make their work possible, regardless of where they worked. There was considerable variation in the reported quality of clinical infrastructure. Doctors in CHAG hospitals complained less about equipment problems than those in public hospitals, and it was doctors in GA (in all types of facilities), who had the most strenuous complaints about lacking necessary equipment, or coping with broken equipment. If we include complaints about over-crowding, GA doctors complained more about infrastructure failings in general than did doctors in BA or UW. While important, this was less a point concerning rural service, per se, other than the fact that inadequacies in equipment in a rural facility could not be addressed through referrals.

Few doctors complained about inadequacies of drug supplies outside GA, but several mentioned emerging problems coinciding with the introduction of insurance, because of delayed and inadequate reimbursements; in a few settings this was identified as leading (for the first time) to inconsistencies in supply, and greater reliance on internally generated funds to ensure adequate stocks.

Discussion of clinical infrastructure prompted several leaders to bemoan the logic of recent investments in higher-quality services such as the new trauma hospital, or an MRI, when so many facilities in Ghana continued to need basic diagnostic equipment, or a "repair and service" culture to ensure quality in basic labs. The continuing reliance on clinical diagnosis of malaria, for example, was cited as emblematic of the need for widespread upgrading of basic facilities, before investing in superior technologies.

#### Schools

Doctors from all regions agreed on the importance of schools if they are to stay in remote areas with their families for long periods. However, this was not generally regarded as an obstacle to solving the problem of rural distribution of doctors; for that, shorter postings were suggested, targeted to younger or older doctors without school-aged children. Many doctors suggested that those without children, or only pre-school children, at the start of their career, would be the best target group for rural service.

"Now I am married and I have a kid of about 2 years, and very soon she will have to be in school. I have been in the region about 5 years, and am planning to go back to further my education, do some postgraduate course.... so hopefully, if everything goes well, I hope very soon I'll be in school, and my kid too, since I would be out of the region and my kid can also get a better place to start her pre-school education." (UW)

#### Ideational Incentives

Religion, political and secular values are some of the ideational factors influencing the self-selection of doctors for rural practice. Notable among these was the invocation of the Christian value of public service as a personal incentive for rural or hardship postings among doctors, as well as explicit mention of the inculcation of socialist values for those trained in the former Soviet Union. The topic of ideological incentives was mentioned most often by professionals in the Upper West.

Some doctors described their appreciation of the respect and notoriety they received from the community for serving in a remote area. It was easy for them to be identified in the area, and their service garnered them a high degree of respect and recognition.

"I don't even look at the money that much; my satisfaction is having the patient walking out of the consulting room with a smile. When a patient comes to my place to say thank you I feel fine; I get more satisfaction than from my salary." (UW)

### Proposed Solutions

The overwhelming majority of doctors, and all leaders, were clear that the MOH needs to institute "pull factors" that will motivate doctors to work in remote parts of the country, and that without such incentives, it is difficult to imagine any improvements in distribution. The need for significant incentives was rationalized by the fact that since doctors are in high demand, they have ample employment opportunities in the private sector, or overseas, and can too easily step out of public service if appointed to a hardship post. At the same time, it was clear that there is an important social prestige afforded to academic and clinical leaders in Ghana, and that this prestige can be exploited as an incentive system. If defined periods of rural service are rewarded with career advancement (e.g. accelerated progression to higher posts), many felt that they would attract more doctors.

Participants were strong and clear about the need to establish reliable reward structures, whereby service for a fixed term in a remote part of Ghana would provide advantages in subsequent appointments, easier admission to a specialist program or foreign study, or preferred access to scholarships.

"If you're in the south and eligible for government-assisted training after two years, here [in the north] you should get it after one year." (Leader)

"First of all they must come out with a system. You come to a deprived area for a maximum of 2 years, and then you have the option to apply for transfer; and there are no conditions imposed, such as finding your own replacement before transfer." (UW)

Leaders frequently argued that service in a hardship post should result in clear, guaranteed advantages, such as faster promotion than for those who stay in the teaching hospitals, since remote service typically results in a more rapid acquisition of skills and experience. Several leaders were looking for ways to provide such training advantages as a means to recruit doctors to their region. One regional director was exploring options with foreign institutions to sponsor special trainings in his region, as a means to attract health staff.

At the same time, doctors in UW and BA, and leaders across Ghana, advocated for mentoring systems that would provide remote doctors with periodic engagement and learning from specialists, programs that could accelerate their learning even faster, while improving the quality of care in rural areas. Almost to a person, doctors in this study were motivated to improve skills and better serve their patients, and most were ambitious to gain recognition for their work.

"They [Ministry of Health] should make sure that once in a while they pay visits; they should let the [doctors] who are in rural areas know that there are people somewhere who think about them and care for them." (UW)

Doctors in GA expressed their motivation to remain in GA because they wanted to specialize early, earn locum, and be part of a dynamic learning environment. Many waxed on about the routine contact with colleagues, especially senior specialists. If mentoring opportunities were re-distributed to remote districts, and career progression actually *favored *rural service, the pull to work outside Accra would likely be stronger among the more ambitious doctors.

There were subtle differences in the articulation of incentive priorities between doctors residing in rural areas and those residing in urban areas. Where as all doctors agreed on the importance of career development, recognition or rewards, mentorship and improved terms of contract, doctors residing in urban areas where more likely to emphasize financial incentives, clear terms of contract and career development. Doctors residing in rural areas were more likely to emphasize career development, clear terms of contract and rewards or recognition. These differences in relative ordering of priority may reflect differences underlying motivational values and ideologies for rural service between doctors residing in rural and urban areas.

Many doctors and leaders advocated for policies to increase the concentration of specialists outside urban areas, suggesting a variety of ways that such policies could attract more doctors to work in the periphery. Those in UW and BA, and many leaders, argued that even occasional access to a specialist would greatly improve motivation for remote doctors. Several leaders proposed that some select remote facilities should have specialists in at least two, if not all four, specialties in order to be accredited for a full two years of housemanship; this, it was argued, would attract a critical mass of young doctors, improving both the learning and the clinical environment.

Several leaders advocated the establishment of learning centers in the north, places where a critical number of specialists would be available for supervision and mentoring. In-service training for doctors is run by Regional HR managers in collaboration with facility directors, so programs can be defined locally. Doctors posted in the surrounding areas could visit for periodic skill-building and refresher courses, enabling them a chance to make contact with senior doctors, and prepare for specialization. Some even suggested that the College should allow specialization while in remote sites, through a system of visiting supervisors.

"...The best approach would be to let [doctors] stay where they are, periodically they can come to the center, have an intensive period of teaching, and go back. Why should people leave their hospitals and travel to Accra? People who have been doing a lot of complicated surgeries in the regions will go to Korle Bu, and they won't even be allowed to do those same operations! It's wasting people's time. Bring them together periodically, and give then assignments." (Leader)

"Surgery is not about reading, reading, it's about a mentor, it's about apprenticeship. Somebody taking your hand and showing you what to do; it's on the job that you learn surgery." (UW)

"Think then again if we can have regular visits from specialists, outreach support to the region to help... and younger ones here can learn a few things from them... that will be an incentive." (UW)

Several doctors and leaders suggested that medical schools could include a compulsory student rotation in rural areas, to alleviate unfounded fears among medical students (often from urban areas), about actual conditions in remote postings.

"They should make sure that there must be a compulsory proposal that in training, or when you finish housemanship, you serve one year there [in remote areas]." (GA)

Leaders were very keen to see an expansion of broadband and computer literacy into the remote areas. Several leaders described how they have promoted internet networks throughout their facilities, and will keep pressing on in this direction. This led to suggestions that they needed more computer literacy for all of their health staff. Leaders were very keen to point out that while many doctors used their computers for personal reasons, they had typically not bridged the divide to professional or workplace applications. Computer literacy classes were identified as an urgent pressing need.

"I think we should encourage every facility to get internet connection, and then introduce them [health staff] to the proper use of internet. We could institute a system of [periodic] conferences and seminars." (Leader)

Finally, leaders clearly wanted a means to delete the names of doctors who don't perform, or sometimes don't even arrive at their posting.

"If you're paid from the public purse, and don't go where I want you, then you better leave and go into private practice. If you're posted to a place and don't go, than [we] should be able to delete your name". (Leader)

"I have 200 staff working under me, and I should have the control over whether they are paid or not, {based on whether} they are working or they are not working. And this is very much needed because they may not do their best, or even come to work, but will still collect their pay. There is no motivation factor. If people do not come to work for one month and you send a message to Accra that they did not work so they should not pay them, they will still pay them. Even people who have vacated their post to go abroad three months, four months ago, they are still getting their pay." (Leader)

The opinion of leaders and practicing doctors in rural areas did not differ remarkably. Both emphasized career development, clear terms of contract and the importance of rewards or recognition. However, regional leaders often had a better appreciation of political levers and the range of feasible incentives, and they more readily debated the pros and cons of various policy options, such as requisite rural credits for specialization, or easier access to specialization or fellowships after a successful period of rural service. Leaders also advocated for compulsory rural rotations with punitive measures for defaulters, and emphasized the need for career opportunities that were integrated with rural services, in order to build clinical capacity through training.

## Discussion

This baseline qualitative study highlights a combination of non-monetary and fiscal incentives for rural service in Ghana, giving prominence to organizational changes focused on career structures [12,13]. Many doctors felt that a short-term post of 1-3 years service in rural areas would be attractive if it was profitable, and especially if one received career benefits for the experience, such as preferential access to educational opportunities.

The importance Ghanaian doctors place on career advancement and the learning environment was consistent with recent findings in Benin, Kenya, and Ghana [8,11], offering policy options beyond monetary incentives. In a study of health worker motivation in Benin and Kenya, qualitative research underscored the importance of further education and professional advancement as a means to motivate both nurses and doctors in the public sector [8]. In Kenya, the prospects for public health sponsorship even made public sectors jobs more attractive than private, despite better working conditions in private facilities [8]. While the current study did not explicitly ask doctors to rate their motivation, the recurring focus on career development as an incentive in these discussions echo the Kenyan findings.

There is no large-scale experimental evidence of using post-graduate training as an incentive for rural service, but the Health Ministry of India launched such an incentive program in 2009. Indian doctors with at least three years of rural service will now be offered a reserved space in a wide variety of postgraduate courses; those with only one or two years of rural service can glean some advantages as well, through a scaled program (10% and 20%, respectively), of added marks towards their entrance application. Such a program offers potential advantages not only for rural health care, but also for strengthening post-graduate training capacity in the country. Expanding opportunities for post-graduate specialization may offer a significant return on investment; establishment of postgraduate training in Obstetrics and Gynecology in Ghana in 1989 led to high retention rates among graduates of the program. Graduates cited the appeal of adding a chance for specialization in their own country to their continued service in Ghana [14].

To increase rural service, and allow it to promote career progression, doctors highlighted several actionable proposals; these included rotations for medical students to increase their rural exposure; establishing CPD credit-earning opportunities outside urban centers; and a targeted policy to increase the number of specialists in regional capitals. Increasing the proximity of specialists in remote regions of the country was emphasized as a way to attract a critical mass of young doctors during housemanship or specialization, enrich the learning environment, and allow rural doctors to gain specialty training themselves. While salary incentives were also proposed, the possibility of organizational incentives is of special importance in this setting given that Ghana increased doctor salaries only three years ago [2,3,15].

Ghana has some history with rural incentive programs, and has progressed from broad to more targeted incentives over the past 2 decades. The largest macro program was the Deprived Area Incentive Scheme/Allowance (DAIA), which targeted 55 deprived districts; each district received an additional monthly allowance of 20-35% above basic salary. The MOH/GHS has not undertaken a systematic evaluation of the DAIA policies, but data from a qualitative evaluation suggested three main public complaints about the scheme: it lacked fairness; it was irregular; and the amounts of added salary were too small to matter. The scheme has since been discontinued.

The Health Staff Vehicle Hire Purchase scheme was initiated in 1997. In 2009, 600 saloon cars of different makes were distributed to health workers; 3494 have been distributed to date. The housing scheme has not progressed very much at national level, however individual agencies have instituted their own schemes. The MOH/GHS has not undertaken a systematic evaluation of any of these policies as of yet.

The findings also underscore the need for increased outreach and communication by the MOH regarding clarity of contract, incentives and postings. The interviews suggest that shorter, defined terms of rural service warrant consideration, a finding consistent with results from Kruk et al that shorter (2-year versus 5-year) contracts in rural hospitals, followed by study leave, were very attractive to senior medical students [11]. Doctors also require greater confidence that the MOH is monitoring their appointments. Frustration and anxiety about the MOH's communication was evident throughout the transcripts - perhaps echoing the high priority placed on "organization and management" in the DCE among medical students [12]. Whether or not the current HR information system is adequate for planning and ensuring timely transfers is unclear, but reliable endpoints appear critical to recruitment. HR information systems are improving rapidly in many countries, including Ghana, offering hope that the technology for centralized HR monitoring in the health sector is not far off. The current scenario is almost a "catch-22" in which weak information systems don't yet offer monitoring data on contract terms, rural recruitment is challenging, and extended rural contracts hinder recruitment all the more. To break such a cycle requires the simultaneous deployment of HR information systems, recruitment incentives, and contracts with reliable endpoints.

The MOH may also want to re-consider how it is managing work-placed accommodation, which appears unsatisfactory for many. In the event that the MOH decides to move towards private-sector housing, with salary compensation to prime the market, infrastructure development will be required in rural areas, as private housing options appeared extremely limited in the north, especially in UW.

There has been significant degree of follow-up to this and several coincident studies in Ghana (one a discrete choice analysis with medical students [11], and a qualitative, in-depth look into the perceptions of health workers in two cities [16]. Senior staff from the Ghana Ministry of Health and the World Bank are co-editing a compilation of studies on human resources in the Ghanaian health sector. Proposed incentives were the topic of policy discussions hosted by the Bank in spring 2010, and at CHARTER Summits in November 2009, and in April 2010. As captured in the Aide Memoire of the 2010 April Health Summit, "the MOH should initiate pilot interventions aimed at improving retention of health workers in deprived/hardship areas based on available evidence".

Follow-up is now within the realm of policy design at the Ministry, and with key development partners. Internationally, many advocate for a mix of fiscal and non-fiscal incentives, but the current evidence in Ghana favors non-fiscal, *organizational *incentives. The lesser emphasis on salaries may reflect the higher salary profile for doctors and nurses in Ghana relative to neighboring countries, and expanding options for professional development.

Physicians appear to have a strong mission to serve clinically, and some aim to re-dress social inequalities, but these coincide with a strong motivation toward specialization and professionalizing themselves in the modern workplace. Clinical specialty training, and professional exposure to learning through new media, telemedicine, or networked data systems: these learning opportunities are mostly still concentrated in Accra or Kumasi. This is amenable to change, however, and the growth of data connectivity across Ghana (and the region) will tremendously enrich any training platform, by providing access to the global library of Open Educational Resources or OER, and Open Access journals.

Policy experiments are expected, including trials to determine whether or not a variety of incentive schemes, evaluated in separate arms over several years, have measureable effects on rural recruitment and retention, the quality of health care, and on health outcomes.

## Conclusions

In the last two 5-year plans [2,3], the Ghana Ministry of Health has proposed a number of incentives for rural service consistent with some of the proposals raised by doctors in these interviews, including a reduced year of service before promotion, a 10% benefit for accommodation, and a free boarding school placement for one child. But these interviews suggest that career advancement incentives will be critical to any successful incentive package. Proposed incentives include guaranteed promotion or study opportunity after service in hardship areas, contact with mentors through rural rotation of specialists or remote learning centers, and reliable terms of appointment with fixed end-points. Such ideas have yet to be piloted in the country; the data generated by this study and that by Kruk [11], offer a basis for re-visiting policy options, and designing trials of select packages.

## Competing interests

The authors declare that they have no competing interests.

## Authors' contributions

RS planned the study, conducted the pilot interviews, contributed to coding and analysis of data, and assumed responsibility for drafting and editing the manuscript. KA contributed to the design and execution of the study, and the coding and interpretation of data. MM and EK conducted the field interviews, contributed to coding, analysis, and writing. KG, MD and JK contributed to the design and execution of the study, and the interpretation of data. MK contributed to the planning and design of the study, interpretation and editing the manuscript. All authors read and approved the final manuscript.
